# Shared TCR Vβ21.3+ T cell immunological signature between MIS-A and MIS-C

**DOI:** 10.70962/jhi.20250050

**Published:** 2026-01-08

**Authors:** Liliane Khoryati, Signe Bech Sørensen, Christophe Parizot, Raphaëlle Lautraite, Marc Pineton de Chambrun, MIS-A & COVID-19 Taskforce, Andreas Ronit, Sofie Eg Jørgensen, Casper Roed, Merete Storgaard, Ann-Britt Eg Hansen, Sarah Benezech, Samira Khaldi-Plassart, Etienne Javouhey, Guillaume Hékimian, Guy Gorochov, Trine H. Mogensen, Alexandre Belot

**Affiliations:** 1 Centre International de Recherche en Infectiologie, Inserm, U1111, Université Claude Bernard, Lyon 1, CNRS, UMR5308, ENS de Lyon, Lyon, France; 2 Department of Biomedicine, Aarhus University, Aarhus, Denmark; 3 Sorbonne Université, Inserm, Centre d’Immunologie et des Maladies Infectieuses, Paris, France; 4 Sorbonne Université-AP-HP, Hôpital La Pitié-Salpêtrière, Département d'Immunologie, Paris, France; 5 Sorbonne Université-AP-HP, Hôpital La Pitié-Salpêtrière, Service de Médecine Intensive Réanimation, Institut de Cardiologie, Paris, France; 6 Department of Infectious Diseases, Aarhus University Hospital, Aarhus, Denmark; 7 Pediatric Rheumatology department, National Reference Centre for Rare Rheumatism, Autoimmune diseases and Interferonopathies, National Reference Centre for Rare Autoinflammatory diseases and Amyloidosis, ERN-RITA, Hospices Civils de Lyon, Lyon, France

## Abstract

Multisystem inflammatory syndrome (MIS) is a severe and potentially life-threatening complication of SARS-CoV-2 infection that affects both children (MIS-C) and adults (MIS-A). While the inflammatory response in MIS-C has been studied in detail, the immune dysregulation underlying MIS-A remains poorly understood, mainly due to the rarity of its condition. Using flow cytometry, we analyzed the T cell response in two, Danish and French, MIS-A cohorts, encompassing a total of 16 cases. We observed an expansion of Vβ21.3+ T cells in at least one of the major T cell subsets (CD3+, CD4+, or CD8+) in 9 out of 16 MIS-A patients. Vβ21.3+ T cells showed increased expression of activation and exhaustion markers along with a higher abundance of effector memory T cells compared to their Vβ21.3-negative counterparts in patients with or without Vβ21.3 expansion. These findings demonstrate that MIS-A shares the same Vβ21.3+ T cell signature previously reported in MIS-C, suggesting a shared pathological immune-related mechanism between the two conditions.

## Teaser

The immune dysregulation in SARS-CoV-2–related multisystem inflammatory syndrome in adults (MIS-A) is not fully characterized. In this work, Khoryati et al. identify a T cell receptor Vβ21.3 signature in MIS-A similar to that previously reported in children (MIS-C), suggesting a shared immune-related mechanism between the two conditions.

## Introduction

In the spring of 2020, case reports emerged describing an unusual severe hyperinflammatory syndrome temporally associated with SARS-CoV-2 in previously healthy children and adolescents (1). This syndrome shared some features with Kawasaki disease but differed in terms of older age (with a median of 8 years) and frequent associations with vasoplegia and prominent gastrointestinal involvement, whereas coronary aneurisms observed in Kawasaki disease were rare (2). This life-threatening condition was named multisystem inflammatory syndrome in children (MIS-C). Although extremely rare, a similar presentation was subsequently reported in adults and termed as multisystem inflammatory syndrome in adults (MIS-A).

As in children, MIS-A onset is estimated to occur 2–6 wk after exposure to SARS-CoV-2. While MIS-A is less common than MIS-C, both conditions share similar clinical and biological features, including persistent fever, systemic hyperinflammation, and multiorgan involvement (3, 4). Particularly, gastrointestinal symptoms and cardiovascular complications are predominantly seen across patients. Severe cases present with multiple organ failure and shock requiring intensive care, but most patients recover with immunosuppressive treatment and supportive care (4, 5, 6, 7).

The pathophysiology of MIS is insufficiently understood. Previous studies and case reports on MIS-C suggested a possible role for inborn errors of immunity (8, 9) and a multitude of immune abnormalities such as elevated levels of innate alarmins and systemic inflammatory proteins, dysregulated innate response, autoantibodies, and aberrant activation of the adaptive response with increased cytotoxicity (10, 11, 12, 13). Specifically, a polyclonal expansion of Vβ21.3+ T cells was observed almost exclusively in MIS-C patients compared to healthy controls, Kawasaki disease, toxic shock syndrome, and mild and severe SARS-CoV-2–infected subjects (13, 14, 15).

To date, and due to the rarity of its occurrence, MIS-A remains poorly studied, particularly at the immunological level. Here, we specifically investigate the T cell response in two European (Danish and French) MIS-A cohorts to determine whether MIS-A shares the characteristic T cell receptor (TCR) Vβ repertoire skewing observed in MIS-C.

## Results

### Clinical and biological features of Danish MIS-A cohort

**Figure 1.**
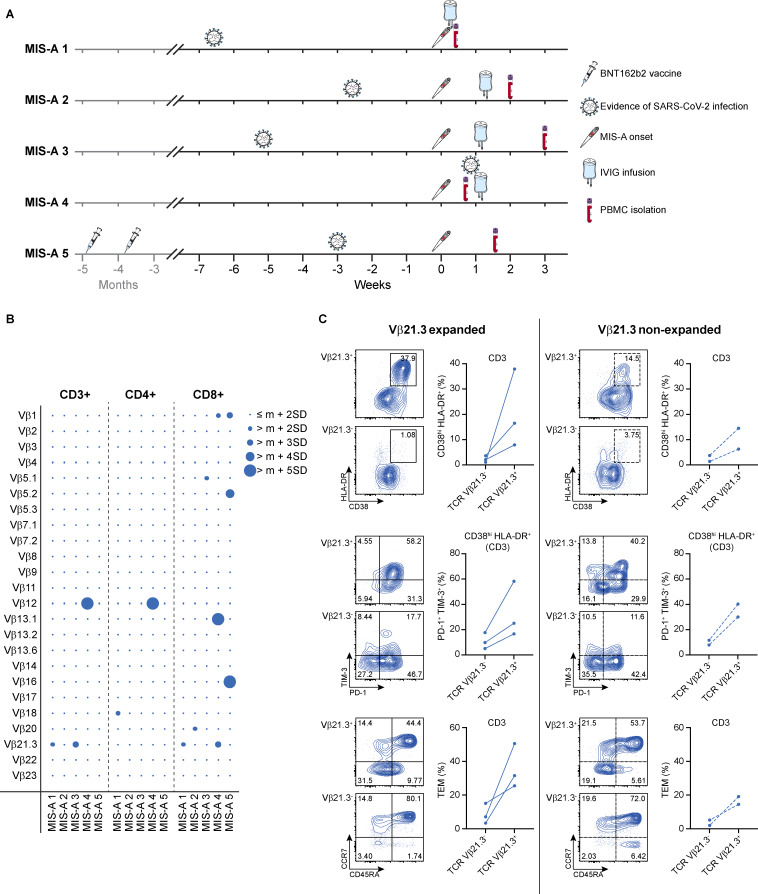
**Expansion and activation of Vβ21.3+ T cells in Danish MIS-A cohort. (A)** Timelines for individual MIS-A patients indicating date of MIS-A onset defined by symptom debut, evidence of SARS-CoV-2 infection, IVIG administration, blood sampling for PBMC isolation, and vaccination. **(B)** Frequency of total CD3+, CD4+, and CD8+ T cells expressing the indicated Vβ chains in Danish MIS-A PBMCs, as measured by flow cytometry. Bubbles represent individual Vβ frequency shown as the standard deviation (SD) from reference mean (m) value for each Vβ subset in the general healthy adult population. Expansion is defined by a frequency greater than the reference mean plus 2SD in healthy population. **(C)** Representative plots and graphical summaries of flow cytometric analyses of Vβ21.3+ versus Vβ21.3− subsets within CD3+ T cells of MIS-A patients. Upper: HLA-DR and CD38 expression on CD3+Vβ21.3+ and CD3+Vβ21.3− T cells. Middle: PD-1 and TIM-3 expression on CD38hiHLA−DR+ subset within CD3+Vβ21.3+ and CD3+Vβ21.3− T cells. Bottom: CD45RA and CCR7 expression defining naïve and memory subset distribution within CD3+Vβ21.3+ and CD3+Vβ21.3− T cells (naïve T cells are CD45RA+CCR7+, central memory T cells [TCM] are CD45RA−CCR7+, EM T cells [TEM] are CD45RA−CCR7−, and EM re-expressing CD45RA T cells [TEMRA] are CD45RA+CCR7−). Graphical summaries of CD45RA−CCR7− TEM frequencies are shown. Plots on the left (solid lines) correspond to MIS-A patients with Vβ21.3 expansion (MIS-A 1, MIS-A 3, and MIS-A 4), and plots on the right (dashed lines) correspond to MIS-A patients without Vβ21.3 expansion (MIS-A 2 and MIS-A 5). Representative contour plots correspond to patient MIS-A 1 (Vβ21.3 expanded) and MIS-A 5 (Vβ21.3 non-expanded).

We first explored a Danish cohort of five MIS-A patients, two males (33 and 50 years old) and three females (23, 38, and 35 years old), referred to as MIS-A 1 through MIS-A 5. All of them were admitted to two major university hospitals in Denmark, between December 2020 and February 2022. Prior to their MIS-A episode, all patients were healthy and had no history of recurrent infections, autoimmunity, or malignancies. All five individuals were infected with SARS-CoV-2 up to 7 wk prior to their hospitalization. Four patients (MIS-A 1–4) were not previously vaccinated against SARS-CoV-2, while MIS-A 5 had received two doses of BNT162b2 mRNA vaccine, 5 and 4 mo before the MIS-A episode (Fig. 1 A).

The patients presented with persistent fever, signs of hyperinflammation, gastrointestinal symptoms, and myocardial dysfunction. Other manifestations included throat irritation, myalgia, severe hypotension, respiratory and circulatory failure, and mucocutaneous involvement. Treatments included intravenous immunoglobulins (IVIGs), corticosteroids, fluid resuscitation, and antibiotics. All patients recovered and were discharged 1 to 2 wk after MIS-A onset. Patients’ characteristics and clinical features are summarized in Table 1.

**Table 1.** Clinical information on MIS-A patients

​	MIS-A 1	MIS-A 2	MIS-A 3	MIS-A 4	MIS-A 5
Age	33	50	23	38	35
Gender	Male	Male	Female	Female	Female
Time from verified SARS-CoV-2 infection to admission	7 wk	3 wk	6 wk	0 day	3 wk
Symptoms from MIS-A period	Persistent fever, dry cough, dyspnea, abdominal discomfort, diarrhea, respiratory and circulatory failure, and universal rash resembling erythema multiforme	Persistent fever, headache, sore throat, aching muscles and joints, abdominal pain, swollen lymph node on left side of neck, universal rash, and respiratory distress	High fever, myalgia, throat irritation, cough, expectoration, retrosternal chest pain, conjunctival injection, chapped lips, and bilateral lung consolidation	Persistent fever, sore throat, diarrhea, hypotension (80/60 mmHg), and myocarditis	High fever, abdominal pain, vomiting, myalgia (especially in lower extremities), labile blood pressure, and multiorgan failure
Treatment	IVIG, hydrocortisone, fluid resuscitation, vasopressor therapy, hemodialysis, ceftriaxone, clarithromycin, and respirator	IVIG, hydrocortisone, fluid resuscitation, Pip/Tazo, meropenem, clindamycin, and metronidazole	IVIG, methylprednisolone, acetylsalicylic acid, penicillin, and ciprofloxacin	IVIG, fluid resuscitation, and Pip/Tazo	Pip/Tazo and clindamycin
Time of blood sampling after first symptoms	2 days	14 days	21 days	5 days	11 days
Treatment before blood sampling	Yes, during sampling	Yes, just prior to sampling	Yes, just prior to sampling	Yes, during sampling	Yes, just prior to sampling
COVID-19 vaccination status prior to MIS-A	No	No	No	No	Yes, vaccinated × 2 (before MIS period)

​	MIS-A 6	MIS-A 7	MIS-A 8	MIS-A 9	MIS-A 10
Age	46	32	39	18	44
Gender	Male	Male	Female	Male	Male
Time from verified SARS-CoV-2 infection to admission	7 wk	5 wk	Unknown	5 wk	6 wk
Symptoms from MIS-A period	Fever, headache, adenomegaly, dysarthria, confusion, and refractory cardiogenic shock	Chest pain, syncope, complete heart block, and cardiogenic shock	Dyspnea and mixed distributive and cardiogenic shock	Fever, abdominal discomfort, diarrhea, universal rash, chest pain, and cardiogenic shock	Fever, diarrhea, abdominal pain, and cardiogenic shock
Treatment	Fluid therapy, vasopressor, inotropes, VA-ECMO, methylprednisolone, IVIG	External cardiac pacing, inotropes, methylprednisolone, IVIG	Fluid therapy, vasopressors, mechanical, ventilation renal replacement therapy, VA-ECMO, methylprednisolone, IVIG	Inotropes, VA-ECMO, methylprednisolone, IVIG	Fluid therapy, inotropes, vasopressors, mechanical ventilation, VA-ECMO, renal replacement therapy, methylprednisolone, IVIG
Time of blood sampling after first symptoms	5 days	6 days	12 days	7 days	1 day
Treatment before blood sampling	Yes, just prior to sampling	Yes, just prior to sampling	Yes, just prior to sampling	Yes, just prior to sampling	No
COVID-19 vaccination status prior to MIS-A	Yes, 3 doses (last dose 10 mo prior)	Unknown	Yes, 2 doses	Unknown	No

​	MIS-A 11	MIS-A 12	MIS-A 13	MIS-A 14	MIS-A 15
Age	22	33	25	25	19
Gender	Female	Male	Male	Female	Male
Time from verified SARS-CoV-2 infection to admission	3 wk	4 wk	7 days	Unknown	4 wk
Symptoms from MIS-A period	Fever, cough, sore throat, headache, diarrhea, conjunctivitis, and cardiogenic shock	Fever, adenomegaly, sore throat, and cardiogenic shock	Sore throat, fever, and myocarditis	Fever, sore throat, chest pain, and cardiogenic shock	Diarrhea, myalgia, dyspnea, and cardiogenic shock
Treatment	Fluid therapy, inotropes, vasopressors, methylprednisolone, IVIG	Fluid therapy, inotropes, vasopressors, methylprednisolone, IVIG	Inotropes, methylprednisolone, IVIG	Inotropes, vasopressors, VA-ECMO, methylprednisolone, IVIG	Inotropes, vasopressors, VA-ECMO, methylprednisolone, IVIG
Time of blood sampling after first symptoms	9 days	3 days	7 days	3 days	5 days
Treatment before blood sampling	Yes, just prior to sampling	Yes, just prior to sampling	Yes, just prior to sampling	No	Yes, just prior to sampling
COVID-19 vaccination status prior to MIS-A	No	Unknown	No	No	No

​	MIS-A 16
Age	16
Gender	Male
Time from verified SARS-CoV-2 infection to admission	Unknown
Symptoms from MIS-A period	Chest pain, headache, fever, abdominal discomfort, and cardiogenic shock
Treatment	Inotropes, methylprednisolone
Time of blood sampling after first symptoms	2 days
Treatment before blood sampling	No
COVID-19 vaccination status prior to MIS-A	Unknown

Pip/Tazo, piperacillin/tazobactam; VA-ECMO, venoarterial extracorporeal membrane oxygenation.

Regarding the patients’ biological features, laboratory results from the first 3 days of hospital admission showed high leukocyte counts and elevated levels of the inflammation marker C-reactive protein. We also found high levels of D-dimer, brain-type natriuretic peptide, and troponin, revealing cardiac involvement, when measured in several patients (Table 2).

**Table 2.** Biological features of MIS-A patients

​	MIS-A 1			MIS-A 2			MIS-A 3			MIS-A 4			MIS-A 5		
Day of hospitalization	1	2	3	1	2	3	1	2	3	1	2	3	1	2	3
Hemoglobin level, mmol/L NR: 8.3–10.5	9.4	8.3	7.3	9.2	9.3	8.3	8.0	7.9	7.9	7.0	5.4	4.4	-	8.1	7.6
Leukocyte count, × 10^9^ cells/L NR: 3.5–8.8	19.1	16.9	14.8	13.4	13.4	12.0	7.2	10.9	19.3	9.5	6.3	7.9	5.7	12.1	13.8
Platelet count, × 10^9^ cells/L NR: 145–390	282	307	290	219	188	160	198	186	246	224	157	231	54	46	35
Plasma creatinine, µmol/L NR: 60–105	135	160	140	91	108	82	67	67	68	59	51	45	59	159	205
C-reactive protein, mg/L NV <10	260	230	200	60	86	110	299	376	410	299	284	286	342.8	414.1	407.2
Ferritin level, µg/L NR: 12–300	-	>2,000	-	-	-	-	-	-	734	233	225	231	-	-	449
Plasma lactate, mmol/L NR: 0.7–2.1	2.0	2.2	3.0	1.6	0.7	0.6	24	27	27	1.2	-	-	-	-	-
D-dimer, FEU/L NV <0.5	2.7	9.9	12	-	0.42	-	-	-	713	-	2.1	2.0	>20	12.2	7.9
Alanine aminotransferase, U/L NR: 10–70	199	2,110	5,080	54	53	-	-	-	672	16	16	38	51	56	61
Brain-type natriuretic peptide, pmol/L NV <10.1	-	1,830	-	-	-	-	-	-	216	-	6,753	-	-	-	8,260
Troponin T, ng/L NV <14	-	483	-	-	-	-	-	-	-	2,253	998	300	-	-	76

​	MIS-A 6			MIS-A 7			MIS-A 8			MIS-A 9			MIS-A 10		
Day of hospitalization	1	2	3	1	2	3	1	2	3	1	2	3	1	2	3
Hemoglobin level, g/dL NR: 13–17.5	10.3	8.2	8.1	12.3	11.8	11.4	10.3	9.1	8.1	12.3	11.4	10.7	12.0	9.0	8.1
Leukocyte count, × 10^9^ cells/L NR: 4.0–10.0	37.7	30.6	24.4	7.2	5.2	6.8	25.6	25.9	13.7	11.8	20.7	20.8	27.7	40.5	36.7
Lymphocytes count, 10^9^ cells/L NR: 1.5–4.00	0.8	1.0	0.5	1.7	0.6	0.7	0.8	1.3	0.6	0.5	-	-	1.3	-	1.2
Neutrophils count, 10^9^ cells/L NR: 2.00–7.50	36.6	28.4	23.2	4.66	4.28	5.7	23.8	24.1	12.6	10.7	-	-	24.9	-	34.6
Platelet count, × 10^9^ cells/L NR: 150-400	138	157	148	264	278	394	212	166	66	177	274	266	184	75	64
Plasma creatinine, µmol/L NR: 59–104	255	228	193	92	82	70	401	312	224	58	49	61	-	727	336
C-reactive protein, mg/L NV <5	320	316	-	-	-	64.5	86.9	175	-	353	-	-	328	314	328
Ferritin level, µg/L NR: 6–34	-	​	​	-	-	-	309	-	-	-	-	-	-	-	-
Plasma lactate, mmol/L NR: 0.5–1	5.4	3.8	2.1	1.3	2.0	1.3	7.8	5.6	1.3	1.9	1.1	2.5	3.2	1.9	0.9
D-dimer, ng/ml	-	-	-	-	-	-	-	-	-	990	-	-	-	-	-
Alanine aminotransferase, U/L NR: 16–35	135	130	146	394	362	333	124	129	119	124	-	86	210	89	82
Brain-type natriuretic peptide, ng/ml	>35,000	>35,000	-	5,819	4,640	-	>35,000	19,031	8,199	5,623	15,575	>35,000	>35,000	>35,000	24,306
Troponin T, ng/L	622	793	-	2,132	-	400	150	119	80.6	155	212	231	413	1,399	380

​	MIS-A 11			MIS-A 12			MIS-A 13			MIS-A 14			MIS-A 15		
Day of hospitalization	1	2	3	1	2	3	1	2	3	1	2	3	1	2	3
Hemoglobin level, g/dL NR: 13–17.5	9.9	7.8	8.2	9.3	9.5	8.8	10.8	10.6	9.5	10	8.7	7.3	13.5	7.6	7.1
Leukocyte count, × 10^9^ cells/L NR: 4.0–10.0	18.9	9.2	7.4	31.9	24.0	23.3	19.5	29.3	25.2	25.3	24.1	41.4	9.5	15.0	27.2
Lymphocytes count, 10^9^ cells/L NR: 1.5–4.00	1.0	1.2	0.9	9.0	-	-	5.7	5.0	-	2.8	-	3.7	1.9	-	-
Neutrophils count, 10^9^ cells/L NR: 2.00–7.50	17.3	7.4	5.9	28.7	-	-	17.6	26.6	-	22.3	-	36.0	10.5	-	-
Platelet count, × 10^9^ cells/L NR: 150-400	193	221	279	195	233	268	244	303	345	198	152	192	125	145	182
Plasma creatinine, µmol/L NR: 59–104	55	52	50	549	473	436	63	65	54	234	115	70	103	86	81
C-reactive protein, mg/L NV <5	142	-	41	450	-	-	263	316	-	411	-	-	311	-	-
Ferritin level, µg/L NR: 6–34	-	-	-	-	-	-	-	-	-	-	-	2,166	-	-	-
Plasma lactate, mmol/L NR: 0.5–1	1.2	1.1	0.9	2.5	0.9	0.5	1.6	1.3	1.4	5.4	2.2	1.9	3.3	4.7	1.9
D-dimer, ng/ml	-	-	-	6,080	-	-	-	-	-	1,490	8,960	-	-	>20,000	>20,000
Alanine aminotransferase, U/L NR: 16–35	27	27	53	18	20	20	61	96	74	38	34	24	60	70	54
Brain-type natriuretic peptide, ng/ml	>35,000	18,492	15,964	>35,000	-	-	5,134	-	5,633	16,717	24,056	-	-	30,061	-
Troponin T, ng/L	320	318	317	714	-	711	622	-	-	-	840	859	-	243	180

​	MIS-A 16		
Day of hospitalization	1	2	3
Hemoglobin level, g/dL NR: 13–17.5	12.0	12.1	11.0
Lymphocytes count, 10^9^ cells/L NR: 1.5–4.00	1.2	-	0.8
Neutrophils count, 10^9^ cells/L NR: 2.00–7.50	14.6	-	20.4
Leukocyte count, × 10^9^ cells/L NR: 4.0–10.0	16.8	15.1	21.6
Platelet count, × 10^9^ cells/L NR: 150–400	405	391	382
Plasma creatinine, µmol/L NR: 59–104	77	64	59
C-reactive protein, mg/L NV <5	-	253.7	-
Ferritin level, µg/L NR: 6–34	-	919	-
Plasma lactate, mmol/L NR: 0.5–1	1.4	1.3	1.5
D-dimer, ng/ml	3,270	-	-
Alanine aminotransferase, U/L NR: 16–35	40	52	231
Brain-type natriuretic peptide, ng/ml	10,488	-	-
Troponin T, ng/L	1,540	590	164

NR, normal range; NV, normal value.

### TCR Vβ repertoire screening in Danish MIS-A patients

**Figure S1.**
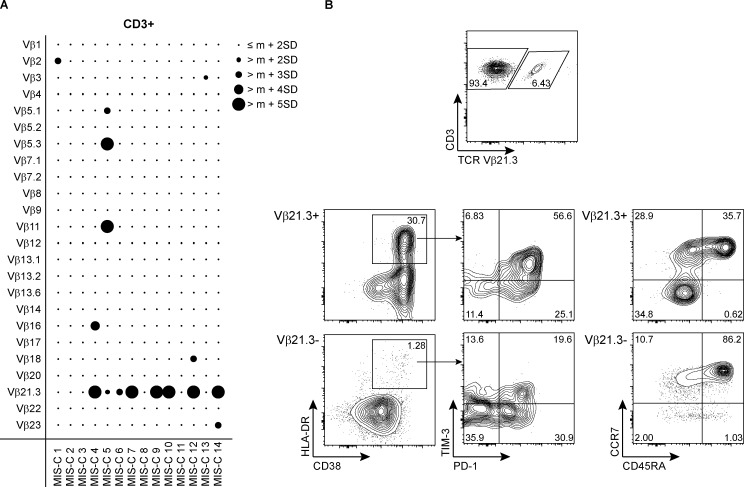
**Expansion and activation of Vβ21.3+ T cells in MIS-C. (A)** Frequency of total CD3+ T cells expressing the indicated Vβ chains in MIS-C PBMCs, as measured by flow cytometry. Bubbles represent individual Vβ frequency shown as the standard deviation (SD) from age-dependent reference mean (m) value for each Vβ subset in the general healthy pediatric population. Expansion is defined by a frequency greater than the reference age-dependent mean plus 2 SD in healthy population. **(B)** Upper: representative plot of Vβ21.3 staining on CD3+ T cells of a MIS-C patient. Middle and bottom: representative plots of flow cytometric analyses of Vβ21.3+ versus Vβ21.3− subsets within CD3+ T cells of the same MIS-C patient, respectively. Expression of HLA-DR, CD38, PD-1, and TIM-3 as well as CD45RA and CCR7 expression defining naïve and memory subsets within CD3+ T cells are shown.

For immunological explorations, we isolated peripheral blood mononuclear cells (PBMCs) from all MIS-A patients within the first month following the beginning of the hyperinflammatory syndrome. Sampling time varied among patients and ranged from 2 to 21 days after MIS-A onset (Fig. 1 A and Table 1). We then analyzed the TCR Vβ repertoire distribution and T cell immunophenotype using spectral flow cytometry. Our results showed an expansion of Vβ21.3+ T cells in at least one of the three T cell subsets, CD3+, CD4+, or CD8+ T lymphocytes, in three MIS-A patients (MIS-A 1, MIS-A 3, and MIS-A 4) (Fig. 1 B). In these individuals, we also found an upregulation of the expression of activation markers CD38 and HLA-DR and of exhaustion markers TIM-3 and PD-1 on Vβ21.3+ cells compared to Vβ21.3− cells within CD3+, CD4+, and CD8+ T cell populations (Fig. 1 C). Additionally, we observed a higher abundance of effector memory (EM) T cells (CD45RA− CCR7−) within the Vβ21.3+ T cell compartment compared to the Vβ21.3− T cells. Together, these data mirror our previous immunological findings in MIS-C (Fig. S1, A and B).

For patients MIS-A 2 and MIS-A 5, from whom PBMCs were harvested at day 14 and 11 after MIS-A onset, respectively, we were not able to detect an overt expansion of the Vβ21.3 family. However, we found a similar immunophenotype of the Vβ21.3+ T cell subset as in Vβ21.3− expanded patients. Specifically, an upregulation of activation and exhaustion marker expression as well as an increase in EM T cells were observed within the Vβ21.3+ population compared to Vβ21.3− cells (Fig. 1 C). Similarly, these data suggest an involvement of the Vβ21.3 T cells in the inflammatory response in MIS-A 2 and 5.

### Confirmation of the Vβ21.3 signature in a French MIS-A cohort

**Figure 2.**
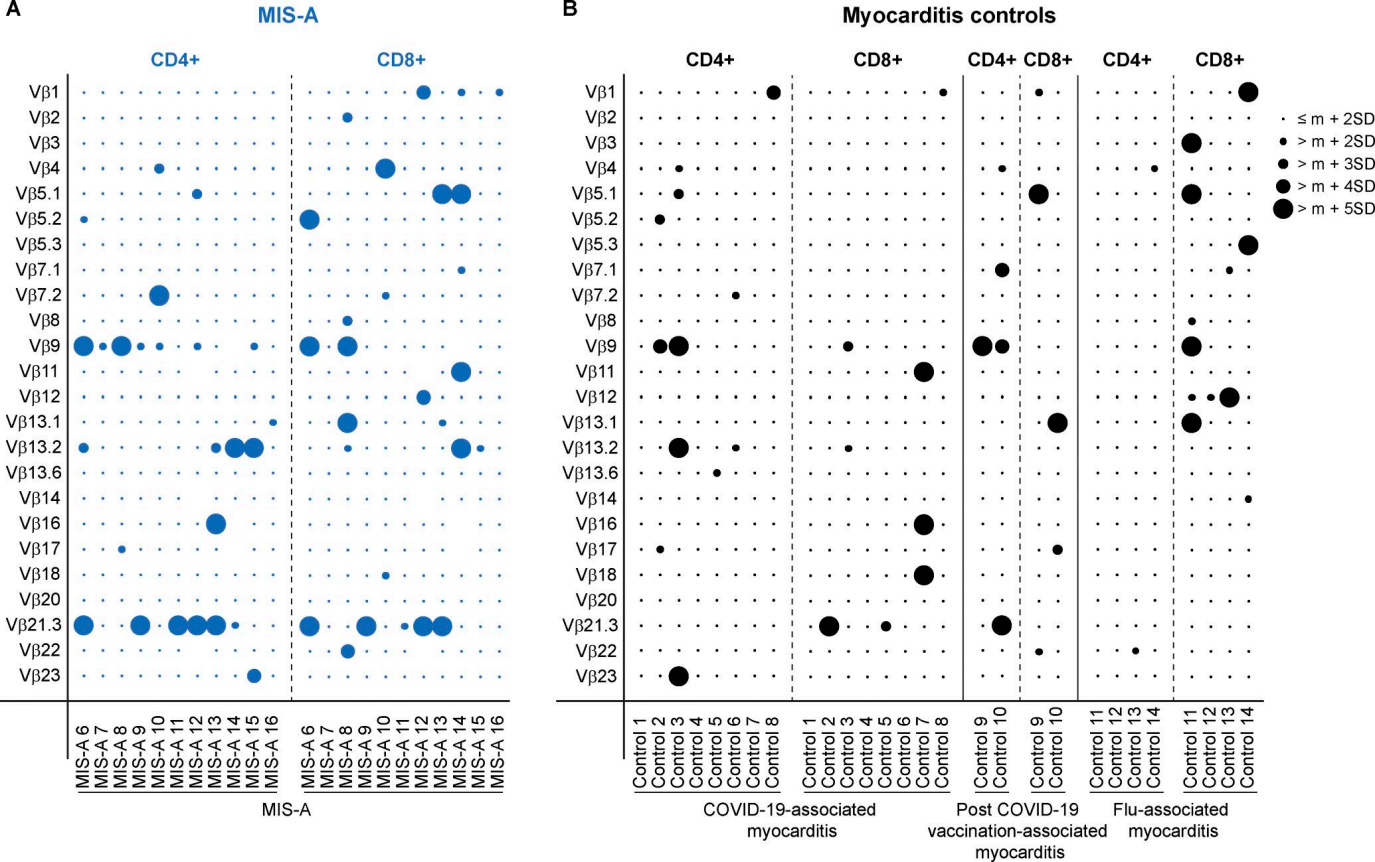
**Confirmation of Vβ21.3+ T cell expansion in French MIS-A cohort. (A and B)** Frequency of total CD4+ and CD8+ T cells expressing the indicated Vβ chains in whole blood from A MIS-A patients and B control groups (COVID-19–associated myocarditis, post COVID-19 vaccination [BNT162b2 mRNA vaccine]–associated myocarditis, and flu-associated myocarditis), as measured by flow cytometry. Bubbles represent individual Vβ frequency shown as the standard deviation (SD) from reference mean (m) value for each Vβ subset in the general healthy adult population. Expansion is defined by a frequency greater than the reference mean plus 2 SD in healthy population. Data for 3 Vβ specificities are missing for MIS-A 12 (Vβ11, Vβ14, and Vβ22) and MIS-A 14 (Vβ9, Vβ16, and Vβ17) due to technical issues while performing the repertoire screening.

We sought to confirm our findings from the Danish MIS-A cases by analyzing the TCR Vβ repertoire in a bigger French cohort of eleven MIS-A patients (referred to as MIS-A 6 through MIS-A 16). For comparison, we included three control groups not meeting the MIS-A case definition criteria: (1) COVID-19-associated myocarditis (*n* = 8), (2) post anti-SARS-CoV-2 vaccination (BNT162b2 mRNA vaccine)–associated myocarditis (*n* = 2), and (3) influenza-associated myocarditis (*n* = 4). All patients were admitted to the intensive care unit (ICU) at the university hospital La Pitié-Salpêtrière in Paris between January 2021 and January 2023. Briefly, prior to their hyperinflammatory episode, all MIS-A patients had a history of SARS-CoV-2 infection (proven by positive nasopharyngeal test or serology), and none of them had received any COVID-19 vaccine. MIS-A clinical symptoms included fever, abdominal and cardiac involvement, hypotension, and shock. Detailed clinical and biological features of these MIS-A patients were previously published (16) and are summarized in Tables 1 and 2. Peripheral whole blood was collected from all patients (MIS-A and controls) upon admission to the ICU, and TCR Vβ repertoire was analyzed by conventional flow cytometry. In line with our results from the Danish MIS-A cohort, we observed an expansion of Vβ21.3+ T cells in CD4^+^ and/or CD8^+^ subsets mainly in MIS-A patients (6/11), and to a much lesser extent in COVID-19–associated myocarditis (2/8) and post COVID-19 vaccination–associated myocarditis (1/2) (Fig. 2, A and B). No Vβ21.3 expansion was detected in the influenza-associated myocarditis group (0/4). Consistent with prior reports in MIS-C (14, 17), we also observed other Vβ expansions in certain MIS-A cases. These expansions, however, were not consistent across patients, with the notable exception of the TCR Vβ9+ subset, which was commonly enriched across all four patient groups (Fig. 2, A and B).

## Discussion

MIS-A is a severe life-threatening complication of SARS-CoV-2 infection in adults and shares with its pediatric counterpart, MIS-C, the same clinical and biological features. Multiple studies characterized the immune dysregulation in MIS-C and highlighted a unique immune signature defined by the expansion of TCR Vβ21.3+ T cells (13, 14, 15, 17). However, the immune response in MIS-A remains poorly explored, mainly due to its exceptionally rare occurrence. Here, we retrospectively investigated the TCR Vβ repertoire in two European cohorts of MIS-A. In the Danish cohort, we found an expansion of Vβ21.3+ T cells in at least one of the three T cell subsets (CD3+, CD4+, or CD8+ T lymphocytes). This expansion was associated with an upregulation of activation and exhaustion markers and an increase in EM T cell frequency, specifically within the Vβ21.3+ T cell population. Interestingly, we also observed an activated profile of Vβ21.3+ T cells in all MIS-A patients, including those lacking Vβ21.3 expansion. The expansion of activated T cell subsets is normally followed by a homeostatic contraction phase during which the frequencies of the expanded populations return back to baseline (18). Consequently, depending on the sampling time from onset of the acute inflammatory episode, skewing of the TCR Vβ repertoire toward a specific Vβ expansion may be attenuated or even lost for late analysis time points. We therefore postulate that the lack of Vβ21.3 expansion in two out of the five Danish MIS-A patients might be related to a delayed timing of sampling after the onset of the inflammatory episode. We then extended our investigations to a larger French cohort where Vβ21.3 expansion was predominantly observed in MIS-A patients, compared to patients affected with infectious or inflammatory myocarditis not related to MIS-A. Of note, no Vβ21.3 expansion was found in influenza-associated myocarditis patients. This observation highlights a difference between SARS-CoV-2 and influenza virus in triggering an inflammatory response in infected subjects. We hypothesize that the differences in viral antigen composition, conformation, or distribution, as well as the nature of the immune response elicited by those antigens, might differentially shape the characteristics of the superantigen-like expansion of T cell subsets. Besides TCR Vβ21.3, we found other Vβ expansions in some MIS-A cases from both cohorts that were not consistent across patients. Such scattered expansions were previously reported in MIS-C, irrespective of concomitant Vβ21.3 expansion (14, 17). We hypothesize that co-expanded Vβ specificities may share homologous binding paratopes with Vβ21.3, and therefore could be activated by similar ligands. Alternatively, these expanded T cells might be responding to different stimuli or reflect bystander activation. Among the MIS-A co-expanded subsets in the French cohort, TCR Vβ9 expansion was almost as frequent as Vβ21.3. However, it should be noted that TCR Vβ9 expansion was also detected in patients from all control myocarditis groups. Endomyocardial lymphocytic infiltrates have previously been reported in myocarditis, associated or not with COVID-19 (19, 20). Thus, it would be of interest to investigate whether Vβ9+ T cells might play a pathogenic role in the myocardial inflammatory process. Finally, it would be important to determine whether, like in MIS-C, Vβ21.3+ T cell expansion in MIS-A is polyclonal, hence suggesting an underlying superantigen effect.

Our study has some limitations, mainly due to its retrospective nature and the rarity of MIS-A cases, which is reflected by the small number of patients in our cohorts. In the Danish one, blood sampling after the onset of MIS-A was performed at different time points among patients and happened only after treatment initiation in three out of five patients. This might have potentially impacted the TCR Vβ repertoire data, particularly by attenuating the observed TCR Vβ skewing. The lack of PBMCs at time of hospital admission hampered our ability to assess the possible effect of treatment administration on TCR Vβ distribution and expanded Vβ specificities in these patients. In the French cohort, we could not perform deep immunophenotyping to assess the activation status of both Vβ21.3+ and Vβ21.3− T cells due to lack of sample availability.

Collectively, the major findings from the present study highlight a skewing of the TCR Vβ repertoire toward a Vβ21.3 response in MIS-A, similar to what was previously reported in MIS-C. Thus, in addition to shared clinical and biological features, the immunological signature of expanded and activated Vβ21.3+ T cells is also common between MIS-C and MIS-A, therefore providing further evidence that these two medical conditions are highly similar and may reflect manifestations of the same immune dysregulation in children and adults, respectively.

Skewing of the TCR Vβ repertoire was very well studied in the context of bacterial and viral superantigens that bypass the antigen specificity of the TCR, thereby inducing a potent polyclonal activation of different T cell Vβ subsets (21). In MIS, the mechanisms underlying the Vβ21.3 response are yet to be determined. Two computational studies suggested the presence of a unique superantigenic motif in the spike protein of SARS-CoV-2 that shares similarities with staphylococcal enterotoxin B and might potentially trigger the polyclonal expansion of Vβ21.3+ T cells and the development of MIS-C (22, 23). However, we have recently unraveled a series of pediatric cases who developed MIS-C in the absence of a SARS-CoV-2 exposure (24). Moreover, a recent study has established a link between TGF-β overproduction and Epstein-Barr virus (EBV) reactivation in MIS-C, suggesting that Vβ21.3+ cells could be EBV specific (25). Further investigations are required to identify potential drivers of the superantigenic-like Vβ21.3 response in MIS-A. Understanding the contribution of Vβ21.3+ T cells to MIS pathophysiology may inform future targeted therapies and help improve clinical management of affected patients.

## Materials and methods

### Patients

This study was approved by the Danish National Committee on Health Ethics (1-10-72-80-20), the Data Protection Agency, and the Institutional Review Board. French cohort study was conducted in accordance with the Declaration of Helsinki using the database registered at the Commission Nationale de l’Informatique et des Libertés (registration no. 1950673), in agreement with the ethical standards of La Pitié-Salpêtrière hospital’s Institutional Review Board, the Committee for the Protection of Human Subjects, and French law.

Danish and French patients were included from two major university hospitals in Denmark (Aarhus University Hospital and Hvidovre Hospital) and the university hospital La Pitié-Salpêtrière in Paris, respectively, between March 2020 and January 2023, after oral and written informed consent were obtained and in accordance with the Helsinki Declaration and national ethics guidelines. Adult patients with a clinical diagnosis of MIS-A could be included in the study if they fulfilled the following criteria (in accordance with Centers for Disease Control and World Health Organization guidelines): documented SARS-CoV-2 infection, prolonged fever, marked inflammation, evidence of clinically severe illness requiring hospitalization, with multisystem (>2) organ involvement, including cardiac involvement and exclusion of other plausible diagnoses, and were treated with a MIS-A–specific therapy. For the Danish cohort, detailed medical history and individual clinical and biological features can be found in the following section and in Tables 1 and 2, respectively. For comparison, MIS-C data provided by previous study (14) were used. For the French cohort, clinical and biological features of MIS-A and control patients were previously published (16) and summarized in Tables 1 and 2.

### PBMC isolation

For PBMC isolation, blood sampling for Danish MIS-A patients was performed between 2 and 21 days after MIS-A onset, as indicated on Fig. 1 A and in Table 1. PBMCs were purified from heparinized blood by Ficoll density centrifugation and stored in liquid nitrogen.

### TCR Vβ repertoire and immunophenotyping by flow cytometry

For the Danish cohort, cryopreserved PBMCs were thawed, washed, and rested for 2 h in complete RPMI medium (RPMI 1640 with GlutaMAX [Gibco] supplemented with 1 mM sodium pyruvate [Gibco], 10 mM HEPES buffer solution [Gibco], 1X penicillin-streptomycin [Gibco], and 10% fetal bovine serum [Eurobio Scientific]). Cells were first incubated with LIVE/DEAD fixable dead cell stain (Invitrogen) for 15 min at room temperature (RT) in PBS (Gibco), then washed, and surface staining antibodies were added for 30 min at RT in PBS supplemented with 0.5% bovine serum albumin (Sigma-Aldrich). Finally, cells were washed and fixed with BD Cytofix/Cytoperm solution (BD Biosciences) for 20 min at 4°C according to the manufacturer’s instructions.

For TCR Vβ repertoire screening, PBMCs (Danish cohort) or whole blood (French cohort) were stained with Vβ-specific monoclonal antibodies from Beckman Coulter (Kit IM3497) according to the manufacturer’s instructions. Vβ expansion was defined by value higher than the mean plus 2 standard deviation in healthy adults (reference values are provided by Beckman Coulter for the Kit IM3497 and are obtained from a cohort of 85 healthy controls except for Vβ4, Vβ7.2, and Vβ13.2 values, which were obtained from 46 controls).

Danish cohort data were acquired on Cytek Aurora spectral flow cytometer and analyzed with FlowJo software (version 10.9.0). French cohort data were acquired on Beckman Coulter DxFLEX conventional flow cytometer and analyzed with CytExpert software (Beckman Coulter, version 2.0.2.18).

### Detailed medical history of Danish MIS-A patients

#### MIS-A 1

MIS-A 1 was a 33-year-old previously healthy Caucasian male who 6 wk after an acute infection with SARS-CoV-2 presented with persistent fever, dry cough, dyspnea, abdominal discomfort, and diarrhea. The patient was admitted to the hospital 1 wk later with a universal rash resembling erythema multiforme and respiratory and circulatory failure, requiring immediate respiratory support, fluid resuscitation, and vasopressor therapy in the ICU. Empirical antibiotic therapy with clarithromycin and ceftriaxone was initiated, together with hydrocortisone.

The patient showed biochemical signs of inflammation, liver injury, and renal failure. A chest x-ray showed bilateral pulmonary interstitial infiltrates. Transthoracic echocardiography (TTE) after intubation showed global hypokinesia (left ventricular ejection fraction [LVEF] ∼20%). Troponin T (TnT) and pro-brain-natriuretic peptide (NT-proBNP) levels were increased. A left upper lobe segmental lung embolus was detected on the chest CT. During admission, the patient was positive for both SARS-CoV-2 antibodies and SARS-CoV-2 PCR oropharyngeal swab. No other underlying etiology was found after extensive microbiological evaluation. Transesophageal echo showed no signs of infective endocarditis.

Hemodialysis was initiated due to renal failure. Simultaneously, the patient was treated with IVIG on suspicion of MIS-A and improved immediately thereafter. The patient was hospitalized a total of 6 days.

#### MIS-A 2

MIS-A 2 was a 50-year-old Chilean man only known with familial hypercholesterolemia. 3 wk prior to admission, he was tested positive for SARS-CoV-2, with mild flu-like symptoms. He was admitted to the hospital 18 days later with fever, headache, sore throat, aching muscles and joints, abdominal pain, swollen lymph nodes on the left side of neck, and a universal rash. Biochemical signs of inflammation were also observed. Contrast-enhanced CT confirmed enlarged cervical lymph nodes and an enlarged left tonsil, suggesting retropharyngeal abscess. This was later disproved by surgical exploration and histological examination of the removed left tonsil.

Initially, empirical piperacillin-tazobactam administration and fluid resuscitation were initiated. This was later switched to meropenem, clindamycin, and metronidazole without any effect. On the fifth day of hospitalization, the patient still had a fever and increased levels of inflammatory biomarkers. He was admitted to the ICU with respiratory distress. Chest-CT showed bilateral infiltrations without signs of pulmonary embolism. TTE showed decreased left ventricular systolic function (LVEF 40–45) without valvular lesions or pericardial effusion. No other underlying etiology was found after extensive microbiological evaluation.

Upon suspicion of MIS-A, the patient was treated with hydrocortisone and IVIG after 8 days of hospitalization. Thereafter, the fever and inflammatory marker levels rapidly decreased, and the patient fully recovered.

#### MIS-A 3

MIS-A 3 was a 23-year-old Caucasian female who tested positive for SARS-CoV-2 with mild lethargy. Approximately 5 wk later, she developed high fever, myalgia, throat irritation, cough, expectoration, retrosternal chest pain, conjunctival injection, and chapped lips. She was admitted to the hospital 1 wk later and initially treated with penicillin and ciprofloxacin for suspected pneumonia.

The patient showed biochemical signs of inflammation, together with increased levels of cardiac enzymes and NT-proBNP. The chest x-ray was normal. Electrocardiogram showed sinus tachycardia with T-wave inversion on all precordial leads. No arrhythmias were detected during telemetry. The TTE was normal. Cardiac MRI showed signs of myocarditis. No other underlying etiology was identified after microbiological evaluation.

On suspicion of MIS-A, IVIG, methylprednisolone, and acetylsalicylic acid were initiated on the third day of hospitalization. Thereafter, the patient recovered quickly.

#### MIS-A 4

MIS-A 4 was a previously healthy, 38-year-old Lebanese woman. She developed fever, sore throat, and diarrhea 6 days prior to admission to the hospital. The patient tested positive for SARS-CoV-2 on hospital admission and presented with persistent fever and hypotension (80/60 mm Hg). The chest x-ray was normal. Biochemical signs of inflammation were observed, together with increased TnT and NT-proBNP levels. TTE showed a decreased LVEF (∼40%).

Initially, piperacillin/tazobactam was administered along with fluid resuscitation. The microbiological evaluation did not identify other causative pathogens. Therefore, IVIG was initiated on the third day when MIS-A was suspected. The fever and inflammation subsided shortly thereafter, and she fully recovered.

#### MIS-A 5

MIS-A 5 was a 35-year-old previously healthy Somali woman who tested positive for SARS-CoV-2 3 wk prior to admission. During hospitalization, she presented with persistent high fever, abdominal pain, vomiting, and myalgia (especially in the lower extremities). The patient had biochemical signs of hyperinflammation, together with increased TnT and NT-proBNP levels. No other underlying etiology was found after microbiological evaluation.

Due to labile blood pressure and multiorgan failure, she was transferred to the ICU. During hospitalization, the patient was treated with piperacillin/tazobactam and clindamycin. She fully recovered.

### Online supplemental material

Fig. S1 shows the expansion and activation status of Vβ21.3+ T cells in MIS-C, thus illustrating the shared Vβ21.3 signature between MIS-C and MIS-A.

## Supplementary Material

Table S1 lists members and affiliations of the MIS-A & COVID-19 Taskforce.

## Data Availability

Raw data underlying main and supplemental figures are available from the corresponding author upon request.
