# Supplementary material for: Shared TCR Vβ21.3+ T cell immunological signature between MIS-A and MIS-C
Source: J Hum Immun. 2026 Jan 8;2(2):e20250050. doi: 10.70962/jhi.20250050 (PMC12871946; doi:10.70962/jhi.20250050)
Supplement: Table S1 — lists members and affiliations of the MIS-A & COVID-19 Taskforce. [file jhi_20250050_tables1.docx]

MIS-A and MIS-C COVID-19 clinicians’ group:

- Andreas Ronit, Department of Infectious Diseases 144, Hvidovre Hospital, University of Copenhagen, Hvidovre, Denmark
- Sofie Eg Jørgensen, Department of Biomedicine, Aarhus University, Aarhus, Denmark
- Casper Roed, Department of Infectious Diseases 8632, Rigshospitalet, University of Copenhagen, Copenhagen, Denmark
- Merete Storgaard, Department of Infectious Diseases, Aarhus University Hospital, Aarhus, Denmark
- Ann-Britt Eg Hansen, Department of Infectious Diseases 144, Hvidovre Hospital, University of Copenhagen, Hvidovre, Denmark
- Sarah Benezech, CIRI, Centre International de Recherche en Infectiologie, Inserm, U1111, Université Claude Bernard, Lyon 1, CNRS, UMR5308, ENS de Lyon, F-69007, Lyon, France
- Samira Khaldi-Plassart, ﻿Pediatric Nephrology, Rheumatology, Dermatology Unit, Hôpital Femme Mère Enfant, Hospices Civils de Lyon, Lyon, France
- Etienne Javouhey, Pediatric Intensive Care Unit, Hôpital Femme-Mère-Enfant, Hospices Civils de Lyon, Bron, France.
- Guillaume Hékimian, Sorbonne Université-AP-HP, Hôpital La Pitié-Salpêtrière, Institut de Cardiologie, Service de Médecine Intensive-Réanimation, Paris.
